# The results of photorefractive keratectomy with Mitomycin-C in myopia correction after 5 years

**DOI:** 10.12669/pjms.321.8576

**Published:** 2016

**Authors:** Masih Hashemi, Mohammad Aghazadeh Amiri, Mehdi Tabatabaee, Ali Ayatollahi

**Affiliations:** 1 Dr. Masih Hashemi, MD, Eye Research Center, Rassoul Akram Hospital, Iran University of Medical Sciences, Tehran, Iran; 2 Dr. Mohammad Aghazadeh Amiri, OD, Department of Optometry, Faculty of Rehabilitation, Shahid Beheshti University of Medical Sciences, Tehran, Iran; 3 Mehdi Taba Tabaee, MSc, Department of Basic Sciences, Faculty of Rehabilitation, Shahid Beheshti University of Medical Sciences, Tehran, Iran; 4 Ali Ayatollahi, MSc, MSc Student of Optometry, Shahid Beheshti University of Medical Sciences, Tehran, Iran

**Keywords:** Photorefractive keratectomy, Myopia

## Abstract

**Objective:**

To evaluate the results of photorefractive keratectomy (PRK) with mitomycin C in myopia correction after five years.

**Methods:**

This is a cross sectional study which included 145 eyes of 74 patients in 18 to 51 years age group that were undergoing Photorefractive keratectomy with mitomycin C using Allegretto Wave Eye-Q 400-Hz excimer laser platform in Markazi Eye Center, Tehran, Iran. All the surgical procedures were performed by the same surgeon. After five years follow-up evaluation including BCVA, UCVA, Refractive error measurement and external eye examination was performed.

**Results:**

The mean diopter of spherical equivalent before surgery was -3.40±1.73. The following findings were obtained after 5 years follow up visit: The mean spherical equivalent value: -0.08±0.40, the mean: Log MARUCVA: 0.02±0.07, the mean Log MAR BCVA: 0.00±0.04.

**Conclusion:**

PRK is an effective, safe and predictable method used to correct myopia. The wave front-optimized algorithm of the Allegretto Wave Eye-Q 400-Hz excimer laser platform demonstrated good refractive and visual results. Presence of variables such as gender, age and astigmatism before operation have no significant impact on the result of this operation.

## INTRODUCTION

Photorefractive keratectomy (PRK) was introduced in the late 1980s, and is the oldest method of surface ablation by Excimer.^1^-^4^ Among the most important Complications that are listed for PRK Corneal haze and myopic postoperative regression can be referred to.^5^-^7^ Many studies have investigated the results of PRK and introduced this procedure as safe, effective, and predictable method to correct myopia.^6^,^8^-^9^

Most studies, especially in cases that used Mitomycin C after surgery, short-term results are investigated and only few studies have examined long-term results of PRK surgery by using Mitomycin C. On the other hand, there are no long-term studies that have investigated the results of this surgery using the Allegretto Wave Eye-Q 400-Hz excimer laser platform (Wave-Light AG, Erlangen, Germany). The new generation of excimer laser system that presumed to omit 4th order aberrations induced by excimer laser ablation by taking some eye variables into account and using pre-planned ablation profiles.^10^,^11^ In our study, the results of PRK with mitomycin C have been investigated by Allegretto Wave Eye-Q 400-Hz excimer laser platform after five years.

## METHODS

One hundred sixteen patients were operated by a surgeon between October 2007 to February 2009 in Markazi Eye Center, Tehran, Iran. Finally from these patients 145 eyes of 74 patients (25 males and 49 females) were examined five years after the date of surgery.

Inclusion criteria for surgery were minimum 18 and maximum age of 40 years, and stable refraction for one year. Patients have been wearing lenses for two weeks before the operation. Patients who did not have prohibition of surgery, after completing consent form using wave front-optimized algorithm of the Allegretto Wave Eye-Q 400-Hz excimer laser platform (Wave-Light AG, Erlangen, Germany) underwent surgery by a surgeon.

Optical zone was chosen based on the amount of refractive error, patient age, and corneal thickness as 6.00 and 6.50 mm. before surgery tetracaine hydrochloride 0.5% eye drops was applied for topical anesthesia. Corneal epithelium was removed by alcohol (20%) and using a hockey knife. At this stage, The Excimer Laser was emitted to surface of Bowman’s membrane and the anterior stroma, and then Mitomycin C solution based on the amount of refractive error was put on the eye and finally washed with sterile water and the band aged contact lens was put on the eye for five days. All patients received Chloramphenicol 0.5% and betamethasone 0.1% after surgery for five days. Betamethasone 0.1% was used four times a day for one times and then three times a day for another month. Patients were examined two days, five days, one month and two months after surgery.

### Preoperative & Postoperative Examinations

All examinations were performed before and after the operation by one person. Preoperative examination included measurement of maximum uncorrected visual acuity (UCVA) and maximum visual acuity using the best corrected visual acuity with the best optical correction (BCVA) using Chart Projector model ACP-31 manufactured by TOPCON, subjective and cycoplegic refraction, pachymetry by Quantel medical device model Pocket 11, investigation of characteristics of the cornea by Orbscan device Manufactured by Baush and Lomb company, slit Lamp examination and evaluation of the retina. Five years after the operation the patients were called and invited to participate in the project and various tests including measurement of UCVA and BCVA using projector chart model ACP-31 manufactured by TOPCON company, subjective and cycoplegic refraction, Slit Lamp examination and retinal examinations were performed.

Finally the data of visual acuity of the Snellen fraction was converted to its equivalent in logMAR system. Variables were compared based on the presence or absence of at least 0.50 diopters of astigmatism, gender and age (two groups less and equal to 25 years at operation time and above 25 years at operation time).

### Statistical Analysis

Data analysis was performed Using SPSS-18 software. For comparing the data before and after operation in normal data Paired T-Test was used and for comparison among groups in normal data T-Test for two independent samples was used. And for non-normal data Mann-whitney U test was used. All statistical tests were performed at α = 0.05 level of error.

## RESULTS

The mean age of 49 women and 25 men who had completed their examination was 29.47±7.1 (range 18 to 51 years old) at the time of surgery. The mean refractive spherical equivalent (SE) before surgery was -3.40±1.73 Diopters (range-0.63 to -9.25 Diopter) and mean LogMAR BCVA before surgery was 0.027±0.09 (range of 0.00to0.46). (Table-I)

**Table-I:** Preoperative characteristics for study group.

Characteristics	No.
No. of eyes	145
Age at time of surgery	29.47±7.10
LogMAR BCVA	0.027±0.09
Sphere (D)	-2.97±1.69
Cylinder (D)	-0.85±0.86
Spherical equivalent (D)	-3.40±1.73

### Refraction

**Fig.1:**
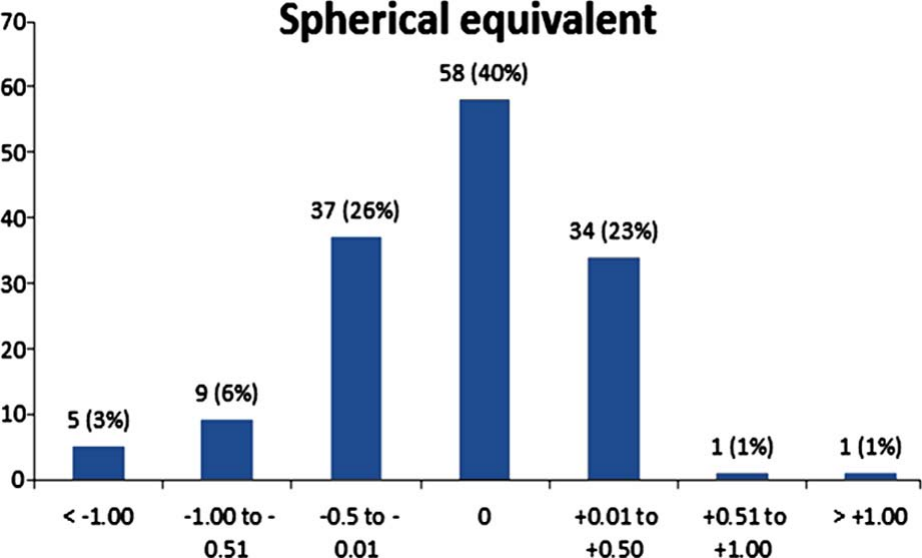
Spherical Equivalent. Percentage of eyes attaining specified level of spherical equivalent 5 years after surgery.

Mean of refractive spherical equivalent from -3.40 diopters before operation got to -0.08±0.4 diopters 5 years after operation (in the range of -1.25 to 1.75 diopters) (Fig.1) (Table-II).

**Table-II:** Refractive error, uncorrected visual acuity and best corrected visual acuity 5 years after surgery.

Characteristics	No.
No. of eyes	145
LogMARUCVA	0.02±0.07
LogMARBCVA	0.008±0.04
Sphere	-0.01±0.39
Cylinder	-0.15±0.26
Spherical equivalent	-0.08±0.40

**Fig.2:**
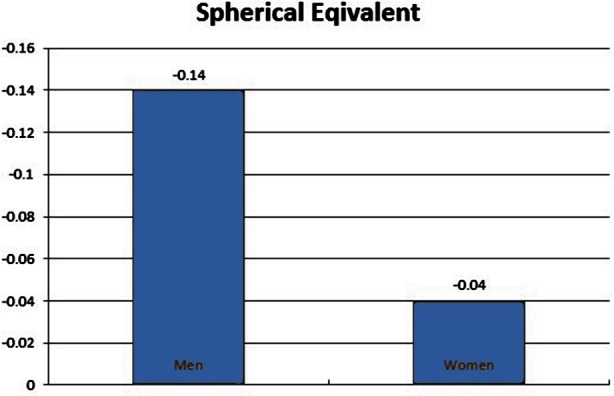
Spherical Equivalent in Men and Women.

Presence of astigmatism before operation and age did not affect postoperative refractive error. But statistically there was a significant difference between two groups of men and women (Pv = 0.05) (Fig.2).

### Visual Acuity

**Fig.3:**
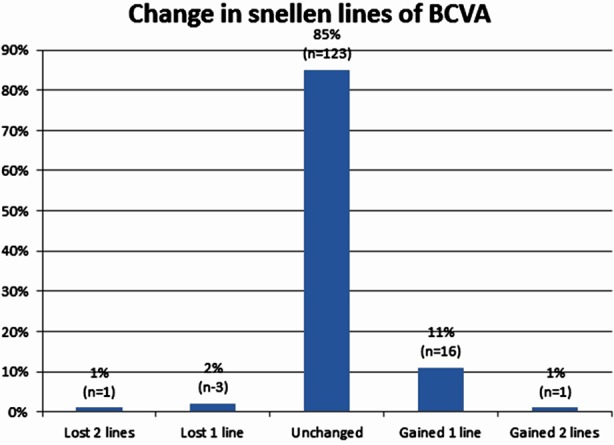
The percentage of eyes that lost or gained Snellen lines of visual acuity 5 years after PRK.

After five years, the mean Log MAR UCVA was 0.02±0.07. Also, 126 eyes (82.2%) obtained UCVA of 20/20. The 136 eyes (93.8%) UCVA of 25/20 or better. Mean Log MAR BCVA was 0.00±0.04. In one eye two Snellenlines and in another 3 eyes 1 line BCVA decreased. Also, in two eyes two lines and in 16 eyes 1 Snellenline BCVA increased (Fig.3) (Table-II). Presence of astigmatism before operation, gender and age did not affect the postoperative UCVA and BCVA.

### Complication

Corneal Haze was not observed in any of eyes during examinations. All corneas examined were clear. Only in one case the amount of refractive error was corrected less than expected that after two years went under second surgery.

## DISCUSSION

In this study conducted in 2015, mean refractives pherical equivalent (SE) before operation was -3.40±1.70 diopters (D). In a study conducted by Jorge L. Alio in 2007, mean SE before operation was -3.81±1.29 D^8^ which is close to our study. But in the study by Shojaei et al in 2007 in Iran, mean SE before operation was -6.15±3.50 D.^6^ Also, in a study conducted in 2013 by Anders H. Vestgaard et al, mean SE was reported as -4.84±2.95 D,^3^ which have investigated myopia higher than our study. It is appropriate to study higher values of myopia in examining the results of PRK by Allegretto Wave Eye-Q 400-Hz excimer laser platform in future. In this study mean SE after operation was obtained as -0.08±0.40 D. In the age group less than & equal to 25 years and more than 25 years, and in both groups with a stigmatism and without a stigmatism before operation there was no significant difference. But there was statistically significant difference between men and women that the difference was clinically 0.09 D which is not noticeable.

In a study conducted in 2013 by Vasilios F. Diakon is et al mean SE in average period of 44.73 months was reported as -0.27±0.70 D.^12^ Also, in the study conducted by Marco Lombardo in 2010 mean spherical refractive error 8 years after surgery was reported as -0.11±0.50 D,^13^ that is close to our study. In these two studies Mitomycin C was used, but in the study done by Vestgaard et al. in 2013, mean SE13to 19 years after surgery was reported as -1.82±1.95 D.^3^ In another study conducted by Norihiko Honda et al in 2004 mean SE 5 years after surgery was reported as -1.11±1.12 D.^14^ The mean SE value in our study was lower than these two studies. Of course it should be mentioned in the aforementioned studies, Mitomycin C is not used and the devices in them included SVS Apex laser system and VISX 20/20, respectively, that are older generation compared to the Allegretto Wave Eye-Q 400-Hz excimer laser plat form and this difference the results of refractive error can be caused by these two issues.

The mean LogMAR UCVA five year after operation was 0.025±0.076 and there was no significant difference between two groups of men and women as well as the age group less than and equal to 25 years and more than 25 years, and two groups with astigmatism and without astigmatism before operation. In a study by Anders H. Vestgaard et al in 2013 by SVS Apex laser system without using Mitomicyne C, mean Log MAR UCVA 13 to 19 years after surgery was obtained 0.16±0.34 which is more than our study.^3^ In another study by Vasilios F. Diakonis et al in 2013 on the Allegretto laser platform 200hz using Mitomycin C, mean Log MAR UCVA 44 months after surgery was reported as 0.04±0.12 that is close to our study.^12^ In this study meanLog MAR BCVA five year after operation was 0.008±0.044 and there was no significant difference in men and women groups as well as the age group less than & equal to25 years and more than 25 years and two groups with or without astigmatism before the operation.

Study conducted by Anders H. Vestgaard et al in 2013, mean LogMAR BCVA after operation was reported as -0.08±0.11 that less and close to our study.^3^ By observing various reports in visual acuity and similarity of BCVA results after operation, it seems that PRK surgery with different devices have little impact on BCVA after surgery and less amount of UCVA after operation in some studies is just because of refractive error and myopic regression After operation.

## CONCLUSION

PRK is an effective, safe and predictable method used to correct myopia. The wave front-optimized algorithm of the Allegretto Wave Eye-Q 400-Hz excimer laser platform demonstrated good refractive and visual results. Presences of variables such as gender, age and astigmatism before operation have no significant impact on the result of this operation. Using Mitomycin C and the new generation machines like Allegretto Wave Eye-Q 400-Hz excimer laser platform has high and positive impact on the results of PRK surgery.
